# Comparison of Modified Natural Cycle Versus True Natural Cycle Frozen Embryo Transfer in Terms of Clinical Pregnancy and Live Birth Outcomes: A Single-Centre Retrospective Cohort Study

**DOI:** 10.3390/jcm15208036

**Published:** 2026-10-16

**Authors:** Gonca Yetkin Yıldırım, İpek Uzaldı, Tuba Varlı Yelke, Nur Cansu Yılmaz Baldan

**Affiliations:** IVF Unit, Department of Obstetrics and Gynecology, Istanbul Medipol University, 34810 Istanbul, Türkiye

**Keywords:** frozen embryo transfer, natural cycle, modified natural cycle, live birth, clinical pregnancy, endometrial preparation

## Abstract

**Objectives:** Natural cycle frozen embryo transfer (FET) has become an increasingly preferred approach for endometrial preparation in ovulatory women. Whether modified natural cycle (mNC) protocols incorporating human chorionic gonadotropin (hCG) for ovulation triggering provide any reproductive advantage over true natural cycle (tNC) protocols remains controversial. This study aimed to compare pregnancy and live birth outcomes between mNC-FET and tNC-FET cycles. **Methods:** This retrospective cohort study analysed 256 frozen–thawed embryo transfer cycles in ovulatory women at a single tertiary centre between January 2014 and December 2024. Of these, 91 cycles used an mNC protocol (mNC-FET group) and 165 cycles used a tNC protocol (tNC-FET group). All transfers involved a single day-5 good-quality or top-quality blastocyst, with identical luteal phase support. Baseline demographic and clinical characteristics, ovarian reserve markers, and embryological parameters were compared between groups. Multivariable logistic regression was performed to identify independent predictors of live birth. **Results:** The mNC-FET and tNC-FET groups were comparable with respect to age (29.88 ± 4.28 vs. 29.96 ± 3.78 years, *p* = 0.880), body mass index (24.86 ± 5.27 vs. 23.73 ± 4.58 kg/m^2^, *p* = 0.145), anti-Müllerian hormone concentration (3.28 ± 2.86 vs. 3.05 ± 1.88 ng/mL, *p* = 0.850), and endometrial thickness (9.76 ± 1.55 vs. 9.32 ± 2.18 mm, *p* = 0.065). The mean number of cryopreserved embryos was slightly higher in the mNC-FET group (5.79 ± 3.47 vs. 5.00 ± 3.47, *p* = 0.050). Embryo quality distribution did not differ significantly between groups (top-quality blastocysts: 69.2% vs. 70.3%, *p* = 0.858). Clinical pregnancy rates were 58.2% (53/91) in the mNC-FET group and 55.2% (91/165) in the tNC-FET group (odds ratio [OR] 1.13, 95% confidence interval [CI] 0.68–1.90; *p* = 0.633). Live birth rates were 46.2% (42/91) in the mNC-FET group and 37.0% (61/165) in the tNC-FET group, without a statistically significant difference (OR 1.46, 95% CI 0.87–2.46; *p* = 0.151). Women who achieved live birth had a significantly higher number of cryopreserved embryos than those who did not (5.91 ± 3.68 vs. 4.86 ± 3.29, *p* = 0.024). In multivariable logistic regression, endometrial preparation protocol was not independently associated with live birth (OR 1.27, 95% CI 0.74–2.19, *p* = 0.394). **Conclusions:** True natural cycle and modified natural cycle protocols did not differ significantly in clinical pregnancy or live birth rates in ovulatory women undergoing single frozen–thawed day-5 good-quality or top-quality blastocyst transfer. hCG-triggered ovulation did not confer a significant reproductive advantage over spontaneous ovulation. However, this does not prove that the two protocols are equivalent, and larger, adequately powered studies are needed given the relatively small sample size.

## 1. Introduction

The use of frozen embryo transfer (FET) has expanded substantially in contemporary assisted reproductive technology (ART) practice due to advances in embryo cryopreservation, refinements in laboratory protocols, and the widespread adoption of elective single-embryo transfer and freeze-all strategies [1]. Compared with fresh embryo transfer, FET allows the endometrium to be prepared in the absence of the supraphysiological steroid concentrations that follow controlled ovarian stimulation, which may impair endometrial receptivity and embryo–endometrium synchrony. FET also reduces the risk of ovarian hyperstimulation syndrome and has been associated with comparable or improved live birth rates, as well as with differences in perinatal outcomes, in selected populations [1,2]. Although multiple endometrial preparation protocols are currently used, there is no clear consensus on the most effective approach [1,3]. Among these, hormone replacement therapy (HRT) with exogenous oestrogen and progesterone, true natural cycle FET (tNC-FET), modified natural cycle FET (mNC-FET), and stimulated cycle protocols are the four most commonly employed strategies [1,3].

Modified natural cycle protocols differ from true natural cycle protocols in that ovulation is triggered pharmacologically with human chorionic gonadotropin (hCG) once the dominant follicle exceeds approximately 17–18 mm, rather than awaiting the endogenous luteinizing hormone (LH) surge [1]. The principal advantage of hCG triggering is greater predictability and scheduling flexibility, since the precise timing of embryo transfer can be planned in advance, reducing intensive ultrasound and endocrine monitoring requirements [1]. Whether this pharmacologic modification of the natural cycle improves reproductive outcomes, however, remains a subject of ongoing debate. Because both protocols rely on a spontaneously selected dominant follicle and an endogenous corpus luteum, the window of implantation would be expected to be timed similarly. However, hCG replaces the endogenous LH surge with a longer-acting luteotropic stimulus, may induce ovulation before the endogenous surge has fully developed, and changes the reference point used to schedule the transfer, all of which could theoretically affect corpus luteum function and embryo–endometrial synchrony [1,4,5]. As the two protocols also differ substantially in monitoring burden and scheduling flexibility, determining whether these physiological differences translate into differences in clinical outcomes is of direct practical relevance.

Several randomized controlled trials (RCTs) have compared endometrial preparation protocols in FET. The MONART trial randomized 1428 ovulatory women to natural, modified natural, or artificial cycle strategies and reported comparable live birth rates (36.6%, 33.4%, and 34.0%, respectively) but a significantly higher cycle cancellation rate in both natural and modified natural cycle groups [6]. A prospective randomized controlled trial conducted in a university setting in Türkiye randomized 145 patients to tNC-FET or mNC-FET and found no difference in clinical pregnancy (58.9% vs. 54.2%, *p* = 0.565) or live birth rates (49.3% vs. 48.6%, *p* = 0.932) [7]. Similarly, Mojtahedi et al. randomized women undergoing FET to natural or modified natural cycle endometrial preparation and reported no significant difference in pregnancy outcomes between the two approaches [8]. Systematic reviews and meta-analyses have similarly failed to demonstrate a significant difference between cycle types. A 2013 systematic review identified 20 articles for analysis and reported no significant differences in clinical pregnancy, ongoing pregnancy, or live birth rates across natural cycle (true or modified) and artificial cycle protocols [3]. More recently, a 2025 meta-analysis pooling 7 randomized trials (n = 1895) concluded that there is no significant difference in live birth rates between modified natural and true natural cycle FET (relative risk [RR] 0.93, 95% confidence interval [CI] 0.78–1.11) [9]. Likewise, a large retrospective cohort of 3478 transfers reported comparable live birth rates between tNC-FET and mNC-FET (31.9% vs. 38.0%, not significant) and identified significantly higher birth rates in both natural cycle protocols than in artificial cycles [10], while a 2025 single-centre retrospective study also showed improved live birth and clinical pregnancy rates with natural cycle approaches compared to other methods [11].

Luteal phase support regimens may further modulate these outcomes. A 2021 systematic review and meta-analysis specifically examined luteal phase support in natural cycle FET (NC-FET) and concluded that progesterone administration is associated with higher clinical pregnancy (odds ratio [OR] 1.48, 95% CI 1.14–1.94) and live birth rates (OR 1.67, 95% CI 1.19–2.36), whereas hCG-based luteal phase support conferred no detectable benefit [12]. A 2021 systematic review further noted that, although current low-quality evidence suggests that natural cycle strategies may be superior to HRT protocols, the existing data remain insufficient to determine which modification of the natural cycle is most effective [4].

Interpretation of available evidence remains complex due to substantial heterogeneity across studies with respect to patient population, embryo stage (cleavage-stage vs. blastocyst), embryo quality grading, single versus double embryo transfer, luteal support regimens, and ovulation monitoring strategies [1,3,9]. Since embryo quality is one of the strongest determinants of implantation and live birth, mixed-quality embryo cohorts may obscure protocol-specific effects [3].

The present study was designed to address some of these limitations by including only ovulatory women undergoing transfer of a single frozen–thawed day-5 good-quality or top-quality blastocyst under a uniform luteal phase support regimen. Unlike most previous comparisons of tNC-FET and mNC-FET, which included mixed embryo stages, double embryo transfers, or heterogeneous luteal support regimens, this design minimizes embryo-related confounding and allows the effect of the ovulation-timing strategy itself to be evaluated more directly. The primary aim was to compare clinical pregnancy and live birth outcomes between tNC-FET and mNC-FET protocols, with an additional aim of identifying factors independently associated with live birth.

## 2. Materials and Methods

This retrospective cohort study was conducted at the Assisted Reproductive Technology Unit of Medipol University Hospital.

### 2.1. Study Setting and Period

Frozen embryo transfer cycles performed between January 2014 and December 2024 were retrospectively reviewed using electronic medical records. The study was designed to compare clinical pregnancy and live birth outcomes between true natural cycle frozen embryo transfer (tNC-FET) and modified natural cycle frozen embryo transfer (mNC-FET) protocols in ovulatory women, with an additional aim of identifying factors independently associated with live birth. Electronic records were available from January 2014, and all eligible cycles from that date onward were screened. However, natural cycle protocols for FET were not used at our centre before 2018; therefore, all eligible tNC-FET and mNC-FET cycles were performed concurrently between 2018 and 2024.

### 2.2. Study Population

#### 2.2.1. Inclusion Criteria

- Women aged 18–42 years at cycle initiation;
- Regular ovulatory cycles (defined as spontaneous menstrual cycles of 25–35 days with mid-luteal progesterone ≥10 ng/mL or documented ovulation by transvaginal ultrasound);
- Frozen–thawed embryo transfer of a single day-5 good-quality or top-quality blastocyst;
- Endometrial preparation by either true natural cycle (tNC-FET) or modified natural cycle (mNC-FET);
- Non-smokers with no alcohol consumption.

#### 2.2.2. Exclusion Criteria

- Cycles prepared with hormone replacement therapy, letrozole, or follicle-stimulating hormone (FSH) stimulation;
- Cancelled cycles (no embryo transfer performed);
- Cycles with missing clinical or outcome data;
- Double embryo transfer;
- Cleavage-stage (day 3) embryo transfer;
- Transfer of poor-quality blastocysts;
- Donor oocyte or donor embryo cycles;
- Preimplantation genetic testing cycles;
- Severe male factor requiring surgically retrieved sperm;
- Recurrent pregnancy loss (≥3 prior losses);
- Congenital uterine anomalies.

#### 2.2.3. Patient Selection Flow

**Figure 1:**
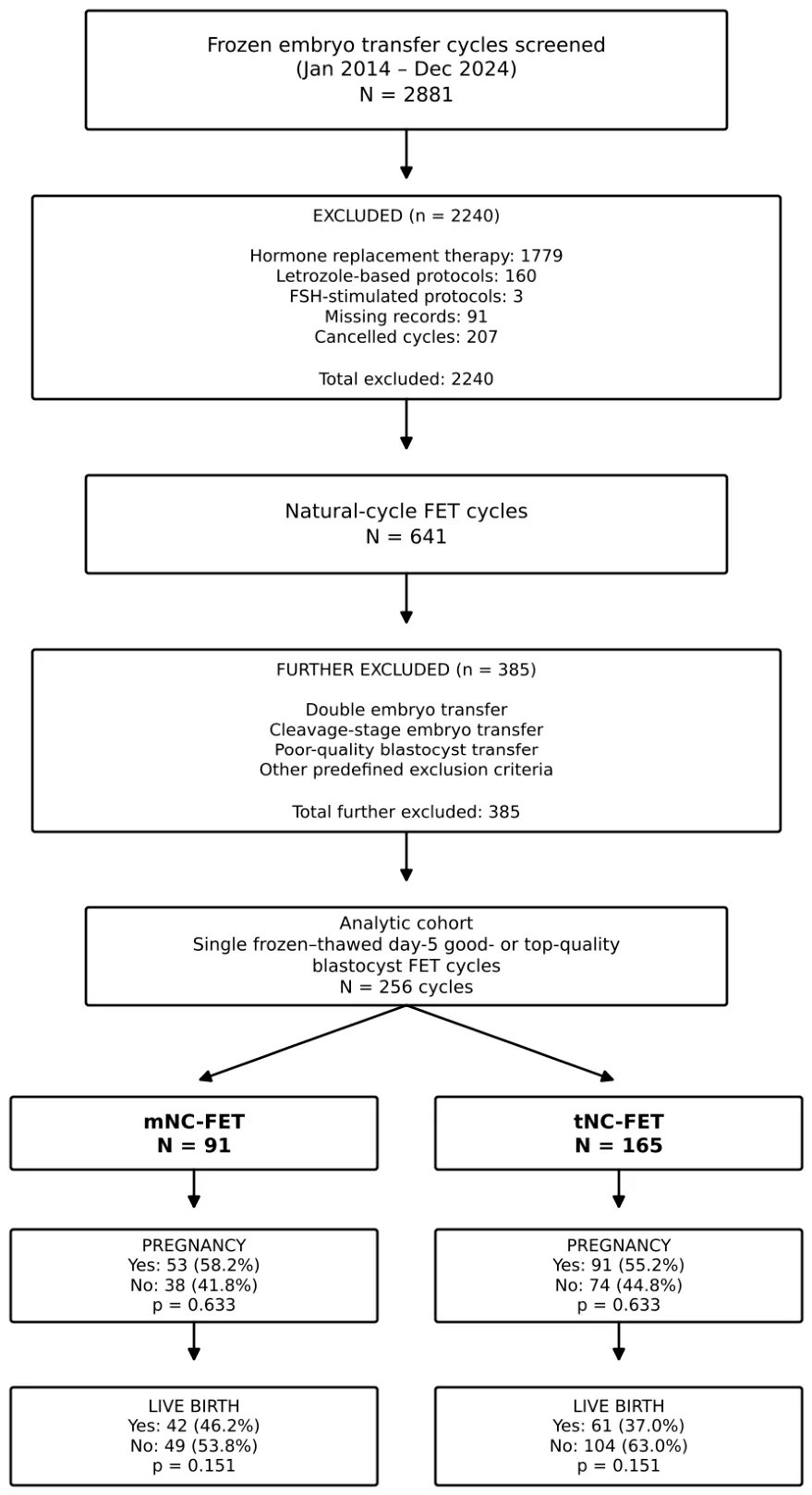
CONSORT-style flow diagram of participant screening, exclusion, and allocation. Of 2881 frozen embryo transfer (FET) cycles screened at the Assisted Reproductive Technology Unit of Medipol University Hospital between January 2014 and December 2024, 2240 were excluded (1779 hormone replacement therapy, 160 letrozole-based protocols, 3 follicle-stimulating hormone (FSH)-stimulated protocols, 91 with missing records, and 207 cancelled cycles), leaving 641 natural-cycle FET cycles. After further exclusion of 385 cycles meeting predefined criteria (double embryo transfer, cleavage-stage embryo transfer, poor-quality blastocyst transfer, and other criteria), the final analytic cohort comprised 256 single frozen–thawed day-5 good-quality or top-quality blastocyst FET cycles—91 performed using the modified natural cycle protocol (mNC-FET) and 165 using the true natural cycle protocol (tNC-FET). Pregnancy was achieved in 53 (58.2%) mNC-FET and 91 (55.2%) tNC-FET cycles (*p* = 0.633), with live birth in 42 (46.2%) and 61 (37.0%) cycles, respectively (*p* = 0.151).

During the study period, a total of 2881 FET cycles were screened. Cycles prepared using hormone replacement therapy (n = 1779), letrozole-based protocols (n = 160), FSH-stimulated protocols (n = 3), cycles with missing records (n = 91), and cancelled cycles (n = 207) were excluded. The remaining 641 natural-cycle FETs were assessed for eligibility. Of these, cycles involving double embryo transfer, cleavage-stage embryo transfer, poor-quality blastocyst transfer, and other predefined exclusion criteria were excluded. The final analytic cohort comprised 256 eligible FET cycles: 91 underwent modified natural cycle frozen embryo transfer (mNC-FET) and 165 underwent true natural cycle frozen embryo transfer (tNC-FET). The patient selection process is presented in Figure 1.

### 2.3. Endometrial Preparation Protocols

#### 2.3.1. True Natural Cycle FET (tNC-FET)

In the tNC-FET protocol, ovulation occurred spontaneously. Cycle monitoring began on cycle days 8–10 with serial transvaginal ultrasound and serum LH, oestradiol, and progesterone measurements [4,13]. When the leading follicle reached a mean diameter of 14–15 mm, monitoring was intensified to alternate-day or daily endocrine and ultrasonographic assessment until ovulation was documented. The endogenous LH surge was defined as a serum LH ≥ 20 IU/L in the presence of a leading follicle ≥ 17 mm and serum oestradiol > 200 pg/mL, consistent with previously published thresholds for natural-cycle FET [1,13]. The day on which the surge criterion was first met was designated LH-Day 0. The day of ovulation was defined as the day following the LH surge and was confirmed by transvaginal ultrasound documentation of follicular collapse (complete disappearance of the follicle or ≥ 50% reduction in follicular volume with collapse of the follicular wall) [14]. Warmed blastocyst transfer was scheduled five days after documented ovulation (ovulation + 5; i.e., LH + 6), consistent with current evidence supporting optimal outcomes in true-natural-cycle FET [4,5].

#### 2.3.2. Modified Natural Cycle FET (mNC-FET)

In the mNC-FET protocol, ovulation was triggered pharmacologically when the leading follicle reached ≥ 17 mm, and the endometrium showed a trilaminar pattern with a thickness ≥ 7 mm, in accordance with the most widely used modified natural-cycle protocol in the literature [4,5]. A single subcutaneous dose of recombinant hCG 250 µg (Ovitrelle; Merck Healthcare KGaA, Darmstadt, Germany) was administered as the ovulation trigger [15,16]. The trigger day was designated hCG-Day 0. Oocyte retrieval was not performed in any cycle. Embryo transfer was scheduled seven days after the hCG trigger (hCG + 7), in line with the most commonly used schedule for blastocyst transfer in mNC-FET [4,5]. In both groups, endometrial thickness was measured by transvaginal ultrasound as the maximal distance between the endometrial–myometrial interfaces in the mid-sagittal plane on the day of LH surge detection (tNC-FET) or hCG trigger (mNC-FET).

### 2.4. Embryo Vitrification–Warming and Quality Assessment

Blastocysts were vitrified and warmed using the Kitazato vitrification–warming protocol (Kitazato Corporation, Shizuoka, Japan) according to the manufacturer’s instructions. Blastocyst quality was assessed on day 5 (120 ± 2 h post-insemination) by at least two experienced embryologists using the Gardner and Schoolcraft grading system, based on expansion stage (1–6), inner cell mass (ICM) grade (A, B, C), and trophectoderm (TE) grade (A, B, C) [17,18]. Good-quality blastocysts were defined as expansion grade ≥ 3 with ICM grade A or B and TE grade A or B (i.e., ≥3BB) [19,20]. Top-quality blastocysts were defined as expansion grade 4–6 with ICM grade A and TE grade A or B (i.e., ≥4AA/AB/BA) [18,20]. Only good- or top-quality blastocysts were eligible for transfer. The number of cryopreserved embryos was defined as the total number of embryos vitrified in the preceding oocyte retrieval cycle and was used as a surrogate of ovarian response in that cycle.

### 2.5. Embryo Transfer

All embryo transfers were performed under transabdominal ultrasound guidance using a soft Wallace catheter (CooperSurgical, Trumbull, CT, USA). With the patient in the lithotomy position and a moderately full bladder, the catheter tip was positioned in the upper-mid portion of the uterine cavity, 1.5–2.0 cm from the fundal endometrial–myometrial interface, in line with previously published optimal transfer-depth data [21,22]. A single good- or top-quality blastocyst was transferred in both study groups. Embryos were loaded using the three-drop technique and expelled under continuous ultrasound visualization, with the position of the air bubble recorded to confirm the transfer depth. The same standardized transfer protocol was used in both groups throughout the study period.

### 2.6. Luteal Phase Support

Luteal phase support was initiated on the day of ovulation in both groups: in tNC-FET cycles, on the day after the LH surge (LH + 1), when ovulation was confirmed by transvaginal ultrasound, and in mNC-FET cycles, on the day after hCG trigger (hCG + 1), corresponding to the expected day of ovulation, as ovulation typically occurs approximately 36–40 h after hCG administration. Thus, the timing of luteal phase support relative to ovulation was equivalent in the two groups. Luteal phase support was continued until 10–12 weeks of gestation in patients achieving an ongoing pregnancy. The regimen consisted of micronized vaginal progesterone (Utrogestan; Besins Healthcare, Brussels, Belgium; 200 mg three times daily) supplemented with oral dydrogesterone (Duphaston; Abbott Biologicals, Olst, The Netherlands; 10 mg three times daily) in both groups, in accordance with the largest body of evidence supporting progesterone-based luteal support in natural-cycle FET [12,23].

### 2.7. Outcome Measures

#### Primary Outcomes

- Clinical pregnancy rate: Defined as the presence of a gestational sac confirmed by transvaginal ultrasound at 6–7 weeks of gestation, calculated as (number of cycles with clinical pregnancy/total number of embryo transfer (ET) cycles) × 100.
- Live birth rate: Defined as the delivery of at least one live-born infant at ≥22 completed weeks of gestation, calculated as (number of cycles resulting in live birth/total number of ET cycles) × 100.

### 2.8. Statistical Analysis

Continuous variables were tested for normality using the Shapiro–Wilk test and are presented as mean ± standard deviation or median (interquartile range) as appropriate. Categorical variables are presented as number (%). Between-group comparisons were performed using the following:

- Independent-samples Student’s *t*-test for normally distributed continuous variables;
- Mann–Whitney U test for non-normally distributed continuous variables;
- Chi-square test or Fisher’s exact test (as appropriate) for categorical variables.

For binary outcomes, unadjusted odds ratios (ORs) with 95% confidence intervals (CIs) were calculated for mNC-FET versus tNC-FET (reference).

A multivariable binary logistic regression analysis was performed to identify independent predictors of live birth. Variables with *p* < 0.20 in univariate analysis and those with established clinical relevance (including age, body mass index (BMI), anti-Müllerian hormone (AMH) level, endometrial thickness, number of cryopreserved embryos, embryo quality, and endometrial preparation protocol) were entered into the model. AMH data were missing for 2 cycles and endometrial thickness data for 3 cycles. Missing data were handled by complete-case analysis (listwise deletion); therefore, 251 cycles with complete data were included in the multivariable model. Results are reported as adjusted odds ratios (aORs) with 95% confidence intervals (95% CIs). A two-sided *p*-value < 0.05 was considered statistically significant. All analyses were performed using IBM SPSS Statistics version 22.0 (IBM Corp., Armonk, NY, USA). A post hoc power calculation was performed using G*Power version 3.1.9.7 (Heinrich Heine University Düsseldorf, Düsseldorf, Germany). Given the final sample size (n = 256; 91 mNC-FET and 165 tNC-FET), this study had approximately 80% power to detect a minimum difference of 15% in live birth rates between groups at α = 0.05.

### 2.9. Ethical Approval

This study was approved by the Ethics Committee of Medipol University (Decision No. E-10840098-50.04-4636; approval date: 7 July 2026). All procedures were performed in accordance with the Declaration of Helsinki. Because the study used only anonymized retrospective data, the requirement for informed consent was waived by the ethics committee.

## 3. Results

### 3.1. Participant Flow

Of the 2881 frozen embryo transfer cycles screened at the Assisted Reproductive Technology Unit of Medipol University Hospital between January 2014 and December 2024, 1779 cycles prepared with hormone replacement therapy, 160 with letrozole-based protocols, 3 with FSH-stimulated protocols, 91 with missing records, and 207 cancelled cycles were excluded, leaving 641 natural-cycle FETs. After stratification, cycles involving double embryo transfer, cleavage-stage embryo transfer, poor-quality blastocyst transfer, and other predefined exclusion criteria were excluded. The final analytic cohort comprised 256 single frozen–thawed day-5 blastocyst FET cycles—91 performed using the modified natural cycle protocol (mNC-FET) and 165 using the true natural cycle protocol (tNC-FET). The patient selection flow is presented in Figure 1.

### 3.2. Demographic and Clinical Baseline Characteristics

Demographic, clinical, and embryological characteristics are summarized in Table 1. The mNC-FET and tNC-FET groups were comparable with respect to mean age (29.88 ± 4.28 vs. 29.96 ± 3.78 years; *p* = 0.880), body mass index (BMI; median 24, interquartile range [IQR] 20.8–28.5 vs. 23, IQR 20.1–26.5 kg/m^2^; *p* = 0.145), serum anti-Müllerian hormone (AMH) concentration (median 2.5, IQR 1.6–4.1 vs. 2.64, IQR 1.72–3.88 ng/mL; *p* = 0.850), and endometrial thickness (median 9.7, IQR 8.6–11 vs. 9, IQR 7.8–10.7 mm; *p* = 0.065). The median number of cryopreserved embryos was marginally higher in the mNC-FET group (5, IQR 3–7 vs. 4, IQR 2–7), approaching statistical significance (*p* = 0.050). The proportion of top-quality blastocysts was comparable between groups (69.2% vs. 70.3%; *p* = 0.858).

### 3.3. Reproductive Outcomes According to Endometrial Preparation Protocol

Pregnancy and live birth outcomes are presented in Table 2. Pregnancy rates were 58.2% (53/91) in the mNC-FET group and 55.2% (91/165) in the tNC-FET group, with no significant difference between protocols (OR 1.13, 95% CI 0.68–1.90; *p* = 0.633). Live birth rates were 46.2% (42/91) in the mNC-FET group and 37.0% (61/165) in the tNC-FET group, and the difference did not reach statistical significance (OR 1.46, 95% CI 0.87–2.46; *p* = 0.151).

### 3.4. Comparison of Baseline Characteristics by Live Birth Outcome

The univariate comparison of baseline characteristics between women who achieved live birth (n = 103) and those who did not (n = 153) is shown in Table 3. The number of cryopreserved embryos was significantly higher among women who achieved a live birth compared with those who did not (median 5, IQR 3–8 vs. 4, IQR 2–6; *p* = 0.024). Mean age showed a trend toward lower values in the live birth group (29.38 ± 4.03 vs. 30.30 ± 3.87 years; *p* = 0.067). Body mass index (*p* = 0.240), AMH (*p* = 0.270), and endometrial thickness (*p* = 0.775) were comparable between outcome groups. Among all 144 pregnancies (58.2% of mNC-FET and 55.2% of tNC-FET patients), 103 (71.5%) resulted in a live birth, while 41 (28.5%) ended in pregnancy loss.

### 3.5. Association Between Maternal Age, AMH, and Cryopreserved Embryo Count

Spearman rank correlation analysis of the entire cohort (n = 256) revealed significant inverse correlations between age and AMH (r = −0.175, *p* = 0.005) and between age and number of cryopreserved embryos (r = −0.241, *p* < 0.001). AMH was strongly positively correlated with the number of cryopreserved embryos (r = 0.410, *p* < 0.001). These correlations were preserved within both the mNC-FET and tNC-FET subgroups.

### 3.6. Multivariable Logistic Regression for Live Birth

A multivariable binary logistic regression model was constructed to identify independent predictors of live birth (Table 4). The model included endometrial preparation protocol, age, BMI, AMH, endometrial thickness, number of cryopreserved embryos, and embryo quality. After adjustment, none of the variables emerged as an independent predictor of live birth. Endometrial preparation protocol was not independently associated with live birth (adjusted OR 1.27, 95% CI 0.74–2.19, *p* = 0.394). The number of cryopreserved embryos showed a borderline non-significant association (adjusted OR 1.08, 95% CI 0.99–1.19, *p* = 0.079), consistent with the univariate analysis.

## 4. Discussion

### 4.1. Principal Finding

In this retrospective cohort of 256 ovulatory women undergoing single frozen–thawed day-5 good-quality or top-quality blastocyst transfer with standardised luteal phase support, modified natural cycle frozen embryo transfer (mNC-FET) and true natural cycle frozen embryo transfer (tNC-FET) yielded comparable clinical pregnancy rates (58.2% vs. 55.2%, *p* = 0.633) and live birth rates (46.2% vs. 37.0%, *p* = 0.151). After adjustment for age, body mass index, anti-Müllerian hormone, endometrial thickness, number of cryopreserved embryos, and embryo quality, endometrial preparation protocol was not independently associated with live birth (adjusted odds ratio 1.27, 95% CI 0.74–2.19, *p* = 0.394).

### 4.2. Comparison with Randomized Evidence

The principal finding aligns with the largest randomized comparison in this field. Ho et al. [6] randomised 1428 ovulatory women to natural, modified natural, or artificial cycle (AC) strategies and reported comparable live birth rates (36.6%, 33.4%, 34.0%) with a higher cycle cancellation rate in both tNC and mNC groups (20.08% each vs. 0% in AC). Aydın et al. [7] prospectively randomised 145 patients and found no significant difference between tNC-FET and mNC-FET in clinical pregnancy (58.9% vs. 54.2%, *p* = 0.565) or live birth (49.3% vs. 48.6%, *p* = 0.932). Likewise, in the RCT by Mojtahedi et al., natural and modified natural cycle FET did not differ significantly in pregnancy outcomes [8]. The numerically higher live birth rate in our mNC-FET group (46.2% vs. 37.0%) did not reach statistical significance; given the limited single-centre sample (n = 256), a clinically relevant difference between the protocols can neither be confirmed nor excluded.

Geysenbergh et al. [24] conducted the largest European multicentre RCT on this topic, randomising 554 ovulatory women across five Belgian centres to natural cycle FET (NC-FET) vs. artificial-cycle FET (AC-FET). They reported clinical pregnancy rates with foetal heartbeats of 33.1% vs. 28.9% (RR 1.15) and significantly lower cycle cancellation in NC-FET (3.9% vs. 9.4%, RR 0.41), consistent with the fact that NC-FET timing anchors to spontaneous ovulation, whereas AC-FET cancellation frequently occurs because of insufficient endometrial thickness or premature spontaneous ovulation. Although Geysenbergh et al. did not directly compare tNC and mNC schedules, the consistency of their clinical pregnancy rate (CPR) comparison with our live birth analysis supports that corpus luteum preservation rather than trigger modality is the principal driver of outcome equivalence. Saupstad et al. [25] recently published the first multicentre, single-blinded RCT specifically designed to optimise the mNC-FET protocol, providing the most contemporary randomised evidence base for our mNC-FET arm.

### 4.3. Comparison with Systematic Reviews and Meta-Analyses

Groenewoud et al. [3] identified 20 articles and reported no significant differences in clinical pregnancy, ongoing pregnancy, or live birth across natural, modified natural, and artificial cycle protocols. Kiose et al. [9] pooled seven randomized trials (n = 1895) and reported no significant difference in live birth between mNC-FET and tNC-FET (RR 0.93, 95% CI 0.78–1.11) but noted substantial inter-study heterogeneity in luteal support regimens, embryo inclusion criteria, and monitoring strategies—limitations the present homogeneous cohort was specifically designed to mitigate. Mümüşoğlu et al. [4] concluded that natural cycle protocols remain valid but emphasised that ongoing RCT evidence is still required.

### 4.4. Comparison with Retrospective Cohorts

Consistent with our results, Bellemare et al. [10] compared protocols in 3478 FET cycles and reported comparable live birth rates between tNC-FET and mNC-FET cohorts. Levi-Setti et al. [26] reviewed 2895 FET cycles (2011–2017), restricted to single blastocyst transfers from vitrified day 5 or 6 embryos, and reported comparable clinical pregnancy and live birth rates across NC-FET, mNC-FET, and AC-FET. The present cohort contributes to this evidence base by restricting the analysis to single day-5 good-quality or top-quality blastocysts in ovulatory women only, thereby removing two major confounders—embryo stage and quality—that have limited prior comparisons.

### 4.5. Role of Luteal Phase Support

Mizrachi et al. [12] examined luteal phase support in natural cycle FET and concluded that progesterone administration is associated with higher clinical pregnancy and live birth, whereas hCG-based support conferred no significant benefit. Greenbaum et al. [27] provided an integrated review and emphasised that luteal phase defect is inherent in non-ovulatory FET cycles and makes exogenous luteal support essential in those settings—whereas in natural cycles, the corpus luteum is generally sufficient and exogenous progesterone corrects only the subset where endogenous secretion falls below the implantation threshold.

Mümüşoğlu et al. [14] investigated patient characteristics predicting low serum progesterone one day before warmed blastocyst transfer in 187 tNC-FET cycles and identified subgroups at risk. Zhang et al. [1] cautioned that luteal phase defect cannot be presumed rare and called for prospective studies defining the optimal progesterone threshold on the day before ET. Our standardised progesterone-based luteal regimen is consistent with current evidence and may partially explain why both groups achieved live birth rates at the upper end of the published range. Finally, because endogenous LH and exogenous hCG activate the same LH/hCG receptor, induce luteinisation and sustain progesterone secretion by the corpus luteum, comparable reproductive outcomes between tNC-FET and mNC-FET are biologically plausible [1,4].

### 4.6. Ovarian Reserve and Live Birth

In our cohort, AMH was strongly correlated with the number of cryopreserved embryos (r = 0.410, *p* < 0.001) but was not associated with live birth in either univariate or multivariable analysis. This finding is biologically plausible: AMH is a quantitative marker of ovarian reserve that predicts the ovarian response and the number of embryos obtained, rather than the implantation potential of an individual embryo. When a good-quality or top-quality blastocyst is available for transfer, the outcome is determined primarily by the developmental competence of the embryo and by endometrial receptivity rather than by ovarian reserve. Similarly, Wyroba et al. showed that in some women with low AMH concentrations, the number of oocytes retrieved, blastocyst formation, and pregnancy outcomes were comparable to those of women expected to respond well, and they emphasised that AMH should not be used alone for prognosis but should be interpreted together with age and other markers [28].

### 4.7. Embryo Morphology

All transfers in the present study involved good-quality or top-quality blastocysts, which minimised the influence of embryo quality on the comparison between protocols. Nevertheless, conventional Gardner grading does not capture all morphological characteristics that may influence implantation. For example, Wyroba et al. reported that oval-shaped blastocysts had euploidy rates similar to those of spherical blastocysts but lower implantation rates in the absence of assisted hatching, a difference that was no longer observed when assisted hatching was performed [29]. Information on blastocyst shape, morphokinetic parameters, and ploidy status was not available in our cohort; therefore, residual heterogeneity in embryo competence cannot be excluded, although there is no reason to expect it to differ systematically between the two protocols.

### 4.8. Clinical Implications

Zhang et al. [1] describe mNC-FET as a trigger-based scheduling approach, contrasting with multi-day serum LH/oestradiol monitoring for tNC-FET. From the patient perspective, mNC-FET reduces repeated venipuncture and ultrasound burden, while tNC-FET avoids exogenous gonadotropin exposure. The Geysenbergh et al. RCT [24] showed NC-FET requires more clinic visits than AC-FET (3.0 vs. 2.4, *p* = 0.0002); a similar trade-off applies between tNC-FET and mNC-FET at our centre.

In our clinical setting, mNC-FET is preferred when scheduling predictability is required; tNC-FET is favoured by patients who wish to avoid exogenous hCG. The Edimiris et al. [30] propensity-matched comparison also suggests programmed ovulatory FET with oral dydrogesterone as a valid injection-free alternative.

Strengths. This study benefits from several methodological strengths. (i) The cohort was restricted to ovulatory women only, eliminating the confounding effect of anovulation and corpus luteum insufficiency. (ii) Only single frozen–thawed day-5 good-quality or top-quality blastocysts were included, removing the dominant confounding effects of embryo stage and embryo grade that have limited prior comparisons. (iii) Both study groups received identical progesterone-based luteal phase support, addressing one of the major sources of heterogeneity identified in the Kiose meta-analysis [9]. (iv) The multivariable logistic regression model adjusted for age, BMI, AMH, endometrial thickness, number of cryopreserved embryos, and embryo quality. (v) The unexpected finding that the median number of cryopreserved embryos differed nearly at the threshold of significance (5 vs. 4, *p* = 0.050) was accounted for in the multivariable model, where it showed only a borderline non-significant association with live birth (aOR 1.08, 95% CI 0.99–1.19, *p* = 0.079). (vi) The Spearman correlation analysis confirmed that AMH and number of cryopreserved embryos are strongly correlated (r = 0.410, *p* < 0.001)—a finding preserved in both mNC-FET (r = 0.515, *p* < 0.001) and tNC-FET (r = 0.371, *p* < 0.001) subgroups.

Limitations. Several limitations must be acknowledged. (i) The retrospective design introduces potential unmeasured confounding despite multivariable adjustment for principal clinical variables. (ii) The sample size (n = 256), although substantial, was not originally powered to detect small absolute differences in live birth rate, and the absence of a statistically significant difference should therefore not be interpreted as evidence of equivalence; the trend toward a higher live birth rate in the mNC-FET group (46.2% versus 37.0%) would require a larger multicentre cohort to resolve definitively, as evidenced by the Levi-Setti 2020 cohort [26], which similarly concluded that subtle protocol-specific differences can only be resolved in sufficiently powered multicentre studies. (iii) The single-centre design at a university hospital ART unit may limit external generalisability to clinics with different stimulation, monitoring, or luteal support protocols. (iv) Secondary obstetric and neonatal outcomes—including miscarriage rate, ectopic pregnancy rate, multiple pregnancy rate, birth weight, and hypertensive disorders of pregnancy—were not the focus of the present analysis and merit dedicated follow-up. (v) The precise luteal support regimen, dose, duration, and route used here should be considered when extrapolating these findings to other institutions. (vi) Although embryo quality was restricted to good-quality or top-quality blastocysts, the precise grading criteria used are those of the institutional embryology laboratory and may differ from those used at other centres. (vii) The tNC group was larger than the mNC group (165 versus 91), reflecting historical practice patterns at our centre, which limits direct matching and may have introduced minor selection bias. (viii) All eligible cycles in both groups were performed concurrently between 2018 and 2024, and laboratory procedures remained unchanged throughout this period (the same sequential culture media, benchtop incubators, blastocyst grading system and Kitazato vitrification–warming protocol were used, and neither assisted hatching nor hyaluronan-enriched transfer medium was used routinely); nevertheless, minor changes in clinical practice over time cannot be completely excluded. (ix) The relatively young age of the study population reflects the strict inclusion criteria, which were limited to non-smoking ovulatory women without alcohol consumption undergoing single day-5 good-quality or top-quality blastocyst transfer; as this selects a good-prognosis population, our findings may not be generalisable to older women or those with diminished ovarian reserve. (x) Detailed data on the preceding oocyte retrieval cycles (ovarian stimulation protocol, total gonadotropin dose, and insemination method) were not consistently available in our retrospective records, and the duration and cause of infertility were not systematically recorded in our database; these variables could therefore not be analysed.

## 5. Conclusions

True natural cycle and modified natural cycle FET protocols did not differ significantly in clinical pregnancy or live birth rates in this cohort of ovulatory women undergoing single frozen–thawed day-5 good-quality or top-quality blastocyst transfer with standardised luteal phase support. hCG-triggered ovulation did not confer a measurable reproductive advantage over endogenous LH-surge-based timing. However, the absence of a statistically significant difference does not prove that the two protocols are equivalent, particularly given the relatively small sample size of this single-centre retrospective study. These findings are consistent with the MONART RCT [6], the Geysenbergh Belgian RCT [24], the Saupstad protocol optimisation RCT [25], the 2025 Kiose meta-analysis [9], and an expanding body of large retrospective evidence [26,30,31].

The choice between tNC-FET and mNC-FET may therefore be guided by scheduling flexibility, monitoring logistics, and patient preference, as no difference in reproductive outcomes has been demonstrated to date. Larger multicentre prospective studies with standardised luteal support regimens remain warranted to confirm these findings and clarify whether the two protocols differ in obstetric and neonatal outcomes.

## Figures and Tables

**Table 1:** Baseline demographic, clinical, and embryological characteristics.

Characteristic	mNC-FET (n = 91)	tNC-FET (n = 165)	*p*-Value
Age (years)—mean ± SD	29.88 ± 4.28	29.96 ± 3.78	0.880 ^†^
Age—median (IQR)	29 (27–33)	30 (28–32)	—
BMI (kg/m^2^)—mean ± SD	24.86 ± 5.27	23.73 ± 4.58	0.145 ^‡^
BMI—median (IQR)	24 (20.8–28.5)	23 (20.1–26.5)	—
AMH (ng/mL)—mean ± SD	3.28 ± 2.86	3.05 ± 1.88	0.850 ^‡^
AMH—median (IQR)	2.5 (1.6–4.1)	2.64 (1.72–3.88)	—
Endometrial thickness (mm)—mean ± SD	9.76 ± 1.55	9.32 ± 2.18	0.065 ^‡^
Endometrial thickness (mm)—median (IQR)	9.7 (8.6–11)	9 (7.8–10.7)	—
Number of cryopreserved embryos—mean ± SD	5.79 ± 3.47	5.00 ± 3.47	0.050 ^‡^
Number of cryopreserved embryos—median (IQR)	5 (3–7)	4 (2–7)	—
Embryo quality, n (%)	—	—	0.858 ^§^
Top-quality	63 (69.2)	116 (70.3)	—
Good-quality	28 (30.8)	49 (29.7)	—

^†^ Student *t*-test; ^‡^ Mann–Whitney U test; ^§^ chi-square test. SD = standard deviation; IQR = interquartile range; BMI = body mass index; AMH = anti-Müllerian hormone.

**Table 2:** Pregnancy and live birth outcomes by group.

Outcome	mNC-FET (n = 91)	tNC-FET (n = 165)	*p*-Value
Pregnancy, n (%)	—	—	0.633 ^§^
Yes (positive β-hCG/clinical)	53 (58.2)	91 (55.2)	—
No	38 (41.8)	74 (44.8)	—
Live birth, n (%)	—	—	0.151 ^§^
Yes	42 (46.2)	61 (37.0)	—
No	49 (53.8)	104 (63.0)	—

^§^ Chi-square test; pregnancy defined as positive serum β-hCG (>10 IU/L) at 14 days post-transfer, with subsequent ultrasound confirmation per institutional protocol. OR = odds ratio; CI = confidence interval. Unadjusted OR (95% CI), mNC-FET vs. tNC-FET (reference): pregnancy 1.13 (0.68–1.90); live birth 1.46 (0.87–2.46).

**Table 3:** Baseline characteristics by live birth outcome.

Characteristic	Live Birth Yes (n = 103)	Live Birth No (n = 153)	*p*-Value
Age (years)—mean ± SD	29.38 ± 4.03	30.30 ± 3.87	0.067 ^†^
Age—median (IQR)	29 (26–32)	30 (28–33)	—
BMI (kg/m^2^)—mean ± SD	24.68 ± 5.18	23.77 ± 4.61	0.240 ^‡^
BMI—median (IQR)	23.6 (20.94–28)	23 (20–26.3)	—
AMH (ng/mL)—mean ± SD	3.34 ± 2.44	2.99 ± 2.15	0.270 ^‡^
AMH—median (IQR)	2.64 (1.73–4.79)	2.5 (1.61–3.5)	—
Endometrial thickness (mm)—mean ± SD	9.44 ± 1.90	9.50 ± 2.06	0.775 ^‡^
Endometrial thickness (mm)—median (IQR)	9 (8.1–10.8)	9.35 (8–10.9)	—
Number of cryopreserved embryos—mean ± SD	5.91 ± 3.68	4.86 ± 3.29	0.024 * ^‡^
Number of cryopreserved embryos—median (IQR)	5 (3–8)	4 (2–6)	—
Group, n (%)	—	—	0.151 ^§^
mNC-FET	42 (40.8)	49 (32.0)	—
tNC-FET	61 (59.2)	104 (68.0)	—
Embryo quality, n (%)	—	—	0.407 ^§^
Top-quality	75 (72.8)	104 (68.0)	—
Good-quality	28 (27.2)	49 (32.0)	—

* *p* < 0.05; ^†^ Student’s *t*-test; ^‡^ Mann–Whitney U test; ^§^ chi-square test; n for individual rows varies due to missing data (AMH n = 151/153, endometrial thickness n = 101/152).

**Table 4:** Multivariable binary logistic regression for live birth (n = 251).

Variable	Adjusted OR	95% CI	*p*-Value
Endometrial protocol (mNC-FET vs. tNC-FET)	1.27	0.74–2.19	0.394
Age (per year)	0.95	0.89–1.01	0.119
BMI (per kg/m^2^)	1.05	0.99–1.10	0.098
AMH (per ng/mL)	1.01	0.89–1.14	0.877
Endometrial thickness (per mm)	0.96	0.84–1.09	0.507
Number of cryopreserved embryos (per embryo)	1.08	0.99–1.19	0.079
Embryo quality	1.10	0.58–2.09	0.768

None of the variables reached statistical significance at α = 0.05. OR = odds ratio; CI = confidence interval.

## Data Availability

The data presented in this study are available from the corresponding author upon reasonable request. The data are not publicly available due to patient privacy and confidentiality considerations.
